# Entropy Scaling of Molecular Dynamics in a Prototypical Anisotropic Model near the Glass Transition

**DOI:** 10.1021/acs.jpcb.3c02429

**Published:** 2023-05-31

**Authors:** Karol Liszka, Andrzej Grzybowski, Katarzyna Grzybowska, Kajetan Koperwas, Marian Paluch

**Affiliations:** Institute of Physics, University of Silesia in Katowice, ul. 75 Pułku Piechoty 1, 41-500 Chorzów, Poland

## Abstract

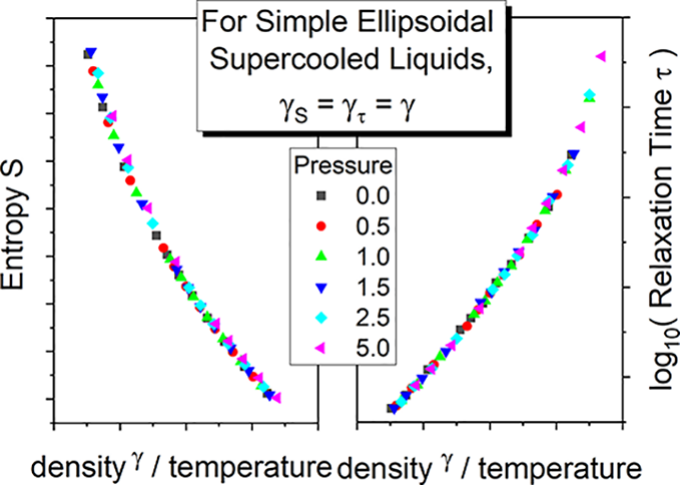

Dynamics and thermodynamics of molecular systems in the vicinity of the boundary between thermodynamically nonequilibrium glassy and metastable supercooled liquid states are still incompletely explored and their theoretical and simulation models are imperfect despite many previous efforts. Among them, the role of total system entropy, configurational entropy, and excess entropy in the temperature–pressure or temperature–density dependence of global molecular dynamics (MD) timescale relevant to the glass transition is an essential topic intensively studied for over half of a century. By exploiting a well-known simple ellipsoidal model recently successfully applied to simulate the supercooled liquid state and the glass transition, we gain a new insight into the different views on the relationship between entropy and relaxation dynamics of glass formers, showing the molecular grounds for the entropy scaling of global MD timescale. Our simulations in the anisotropic model of supercooled liquid, which involves only translational and rotational degrees of freedom, give evidence that the total system entropy is sufficient to scale global MD timescale. It complies with the scaling effect on relaxation dynamics exerted by the configurational entropy defined as the total entropy diminished by vibrational contributions, which was earlier discovered for measurement data collected near the glass transition. Moreover, we argue that such a scaling behavior is not possible to achieve by using the excess entropy that is in excess of the ideal gas entropy, which is contrary to the results earlier suggested within the framework of simple isotropic models of supercooled liquids. Thus, our findings also warn against an excessive reliance on isotropic models in theoretical interpretations of molecular phenomena, despite their simplicity and popularity, because they may reflect improperly various physicochemical properties of glass formers.

## Introduction

Despite many decades of research on the glass transition and related phenomena, a proper relation between molecular dynamics (MD) and thermodynamics of the systems approaching the glassy state is still hotly debated. One of the most inspiring aspects of these investigations has been the role of entropy in the thermodynamic evolution of MD timescale at least since 1965 when Adam and Gibbs (AG) formulated^1^ their model linking changes in the MD timescale on varying thermodynamic conditions to those in the configurational entropy, *S*_conf_. The AG conception sparked off a discussion about the proper estimation of *S*_conf_ in the case of molecular glass formers, which has been concluded in the most commonly accepted way by Johari,^2^ who suggested that *S*_conf_ is the difference between the total system entropy of the melt (*S*) and the vibrational contribution to the entropy (*S*_vib_) rather from the glass than from the crystal. Over the last decades, experimental achievements of the high-pressure measurements of supercooled liquids have issued a challenge^3−5^ to the theoretical ideas earlier usually limited to the temperature effect on MD near the glass transition. The investigations of the dynamic and thermodynamic properties of supercooled liquids as functions of temperature *T* and pressure *p* (or alternatively, density ρ = *f*_ρ_(*T*,*p*)) have enabled us to gain a new insight into the relation between MD and thermodynamics of glass formers. Among other things, based on the analyses of measurement data, we have shown that the structural relaxation times τ can be scaled with *S*_conf_,^6^ which complies with our temperature–volume extension^7^ of the originally temperature-dependent MYEGA model^8^ that was formulated by developing the AG model in terms of the constrain theory^9^ and the analysis of energy landscape.^10^ On the other hand, based on MD simulations mainly in the Kob–Andersen binary Lennard–Jones (KABLJ) liquid model,^11^ which is a simple prototypical isotropic model of supercooled liquid, Dyre et al.^12,13^ showed that the structural relaxation times τ can be scaled with the excess entropy *S*_ex_, which is in excess of the ideal gas entropy *S*_id_. This approach has made an attempt at finding a substitute for the configurational entropy, which cannot be evaluated based on such models that do not involve vibrations like the KABLJ liquid. In addition, invoking the isomorph theory,^14^ which was developed and verified mainly by exploiting simple simulation models, the attempt of the excess entropy scaling of τ has been even extended to some experimental data,^12^ although we earlier gave evidence that the structural relaxation times cannot be generally scaled with *S*_ex_ by analyzing measurement data for several glass formers belonging to various material groups.^15^ Thus, an essential question arises as whether there are some simple models that are able to better reflect the experimental results in simulations, and consequently shed a new light on the relationships between MD timescale and entropy. In this paper, we successfully answer the question and show how molecular interactions affect the entropy scaling.

## Simulation Model and Computational Methods

A promising candidate for proper reflecting and predicting the physicochemical properties of supercooled liquids could be a simple model of a well-defined anisotropy of molecular shapes and intermolecular interactions, which would give an opportunity to achieve the glass transition in the MD simulations. Angell and co-workers^16^ showed that the well-known Gay–Berne (GB) ellipsoidal model^17^ that satisfies the expected anisotropic characteristics can be used to simulate the supercooled liquid state and the glass transition at zero pressure. Very recently, we have confirmed that the single-component GB liquid can be supercooled and vitrified also at elevated pressure.^18^ What is more, the GB supercooled liquid systems have obeyed the power density scaling law, τ = *f*_τ_(ρ^γ^/*T*), with a constant value of the scaling exponent γ for a given anisotropy aspect ratio *a*_r_ = (ellipsoid length)/(ellipsoid width), which was successfully tested for *a*_r_ = 1.30, 1.35, 1.40, and 1.45 by using both the translational and rotational relaxation times (τ and τ_rot_) determined from the MD simulations of 1000 ellipsoidal particles in the isothermal–isobaric (*NpT*) statistical ensemble along several isobars (*p* = 0, 0.5, 1.0, 1.5, 2.5, and 5.0 in LJ units). Therefore, the GB supercooled liquid very well comes up to the expectations of implementing a robust model to verify the entropy scaling behavior near the glass transition. It should be noted that we have established^18^ the translational relaxation times τ in the GB model from the time-dependent incoherent selfscattering function on the assumption that *F*_s_(*t*) = e^–1^ at *t* = τ, which is typically employed also in the analyses of simulation data collected in simple isotropic models of supercooled liquids, exploiting its definition, , where **q** is the wave vector at the first maximum of the static structure factor, **r**_*i*_ and **r**_*j*_ indicate centers of mass for particles *i* and *j*, and the brackets < > denote the ensemble average of *N* particles. In this paper, we express all considered quantities in LJ units, which are the standard units in the GB simulation model. However, the isomorph theory units are neglected in our analyses, which is explained the next Section.

To follow the thermodynamics procedure for calculating the total system entropy *S* by using the measurement pressure–volume–temperature (*pVT*) data and the experimental isobaric heat capacity *C*_p_ at ambient pressure,
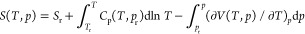
1the total system entropy at a reference state, *S*_r_ = *S*_r_(*T*_r_,*p*_r_) = *S*(*T*_g_(*p*_0_),*p*_0_), has been assumed at the glass transition temperature *T*_g_ at zero pressure *p*_0_ = 0 to analyze the simulation data collected in the GB model. Such a reference state is often used in the case of glass-forming liquids (but at ambient pressure) instead of the melting point. To achieve a high-quality integration over pressure in eq 1, we exploit an equation of state originally derived to explore the volumetric data of supercooled liquids,^19−21^ which has been parametrized by using the *pVT* simulation data for the GB model at each examined anisotropy aspect ratio (eq 3 and Table 1 in ref (18). Other details of the calculations of total system entropy values are presented in the Supporting Information.

In addition, the thermodynamic formula for *S* given by eq 1 requires evaluating the temperature dependence of the heat capacity *C*_p_ at least at zero pressure in the case of the GB model. However, the heat capacity data, which are measured relatively easily by means of the calorimetric techniques, are nontrivial to evaluate from simulation data in simple models in order to properly reflect the temperature dependences of *C*_p_ near *T*_g_ obtained, e.g., from the differential scanning calorimetry measurements.^22^ For instance, the heat capacity values reported^23^ for the KABLJ model considerably increased with decreasing temperature, and any step-like pattern in the temperature dependence of heat capacity, which is characteristic for the glass transition, was not observed. To overcome this problem, we have performed herein additional MD simulations in the GB model near the glass transition at zero pressure in the GB model of 1000 ellipsoids in the *NpT* ensemble in an analogous way to that reported in ref (18), but covering the temperature range every Δ*T* = 0.05 between the temperatures 0.1 and 0.9 at zero pressure, and we applied an accurate averaging every 200 simulation time steps Δ*t* = 0.001 to determine the isobaric heat capacity *C*_p_ from the variance of enthalpy *H*, that is by using the typical method for evaluating *C*_p_ = (<*H*^2^> – <*H*>^2^)*T*^–2^*k*^–1^ from MD simulations,^24^ where the Boltzmann constant *k* = 1 in the LJ units and the brackets < > denote the ensemble average.

## Results and Discussion

**Figure 1:**
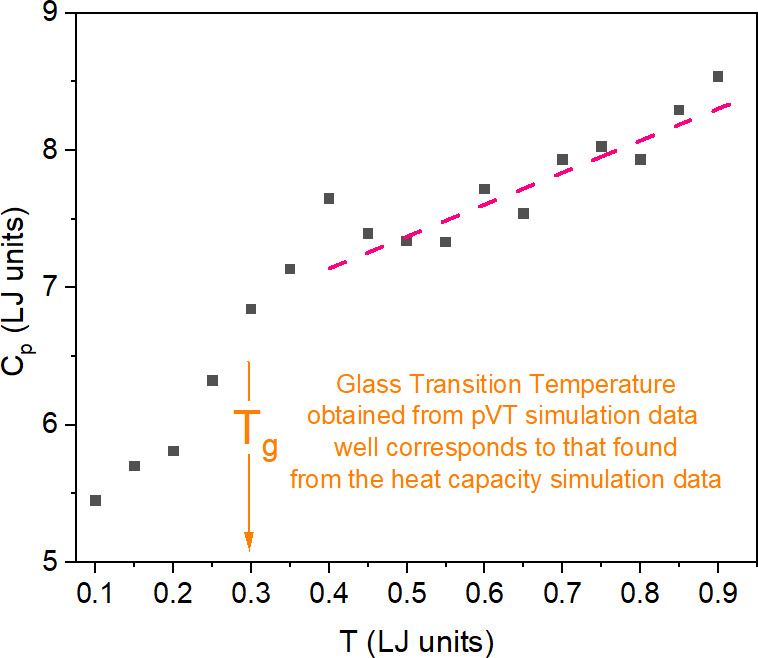
Plot of the isobaric heat capacity *C*_p_ per molecule versus temperature in the GB model at zero pressure for *a*_r_ = 1.30. Dashed line is a linear approximation of the dependence *C*_p_(*T*) in the supercooled liquid state. Values of its parameters are listed in Table 1.

From the viewpoint of MD simulation techniques, our analysis of the additional simulations has made progress in evaluating the heat capacity based on simple models, because we have been able to reproduce the step-like pattern in the temperature dependence of *C*_p_ at the glass transition as shown, e.g., for the anisotropy aspect ratio *a*_r_ = 1.30 in Figure 1. Since the dependences *C*_p_(*T*) measured near the glass transition have typically been approximated linearly in both the supercooled and liquid states, for instance, to calculate the integral over temperature in eq 1 in the case of supercooled liquid, we also analyze the temperature dependences of *C*_p_ determined from our MD simulations in the GB model in such a manner, but only in terms of the region of supercooling.

**Figure 2:**
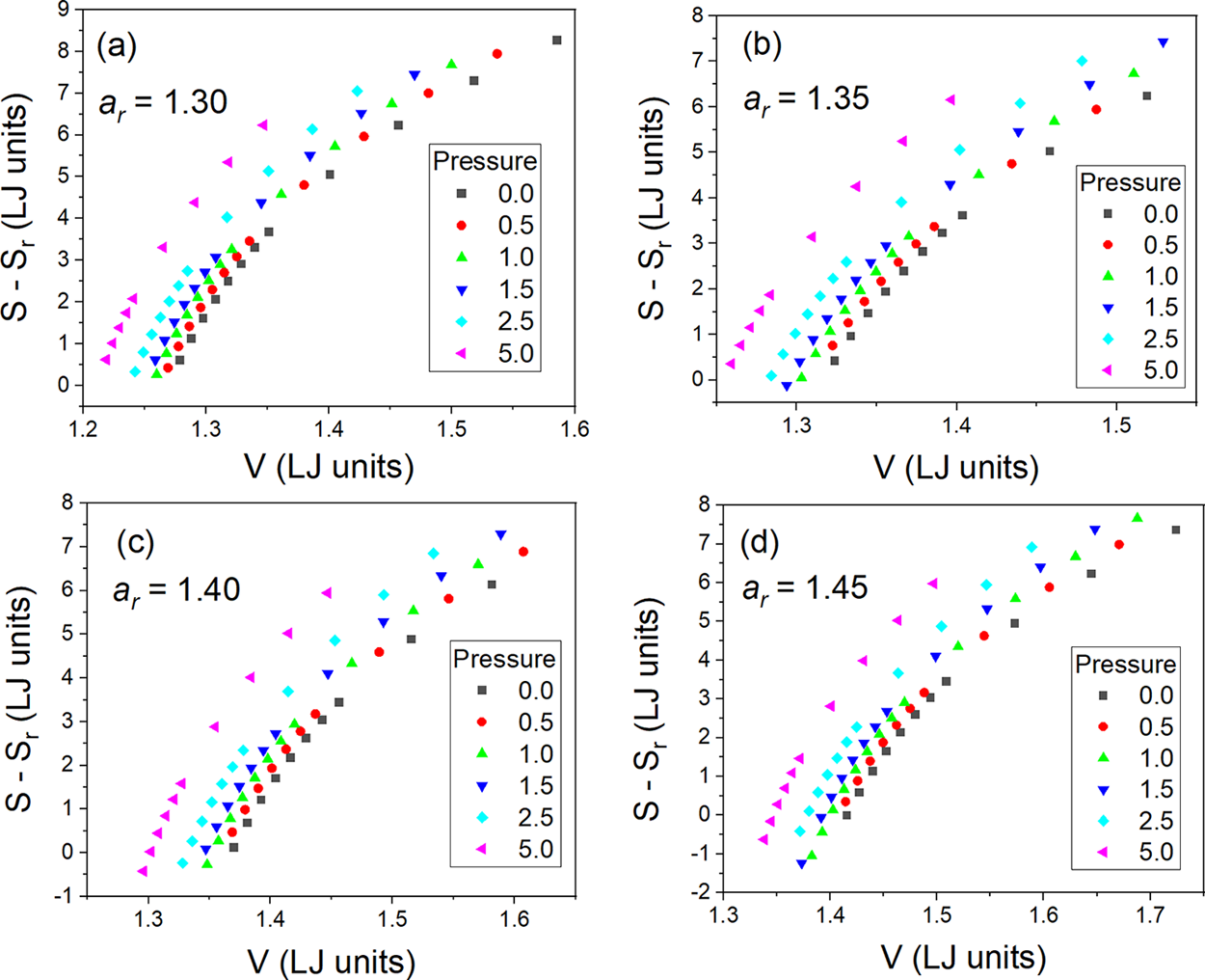
Plots of the total system entropy *S* evaluated from eq 1 by using simulation data collected in the supercooled liquid state of the GB model in several isobaric conditions (at pressure *p* = 0, 0.5, 1.0, 1.5, 2.5, and 5.0 in LJ units) and four different anisotropy aspect ratios *a*_r_ = 1.30, 1.35, 1.40, and 1.45 shown in panels (a), (b), (c), and (d), respectively. *V* denotes the particle number volume, whereas *S*_r_*= S*(*T*_g_(*p*_0_),*p*_0_) at zero pressure *p*_0_ = 0.

The values of parameters of the linear approximations, *C*_p_(*T*) = *C*_1_*T* + *C*_0_, for all examined *a*_r_ at zero pressure in the supercooled liquid state are collected in Table 1, which is sufficient to determine the integral with respect to temperature in eq 1. By using the values of the parameters collected in Table 1 for the dependence *C*_p_(*T*) at zero pressure and the values of the parameters of the equation of state found by fitting the *pVT* simulation data in the GB model to eq 3 in ref (18), which have listed in Table 1 in ref (18), we have found the total system entropy according to eq 1 for each examined anisotropy aspect ratio *a*_r_ at all state points at which the translational and rotational relaxation times have been previously established in ref (18) for the GB supercooled liquid systems. The obtained values of the total system entropy *S* for all examined anisotropy aspect ratios are presented as functions of the particle number volume *V* in Figure 2, which qualitatively very well reproduce such dependences found from eq 1 for measurement data of glass formers, for instance, reported in ref (15).

**Table 1:** Values of the Parameters *C*_1_ and *C*_0_ of the Linear Approximations (*C*_p_(*T*) = *C*_1_*T* + *C*_0_) Found for the Temperature Dependences of the Isobaric Heat Capacity *C*_p_ in LJ Units per Molecule Obtained from the MD Simulations in the GB Model for all Examined Anisotropy Aspect Ratios *a*_r_ in the Supercooled Liquid State at Zero Pressure

*a*_r_	*C*_1_	*C*_0_
1.30	2.33 ± 0.32	6.21 ± 0.21
1.35	2.04 ± 0.32	6.59 ± 0.21
1.40	1.66 ± 0.47	7.02 ± 0.33
1.45	1.30 ± 0.47	7.49 ± 0.33

**Figure 3:**
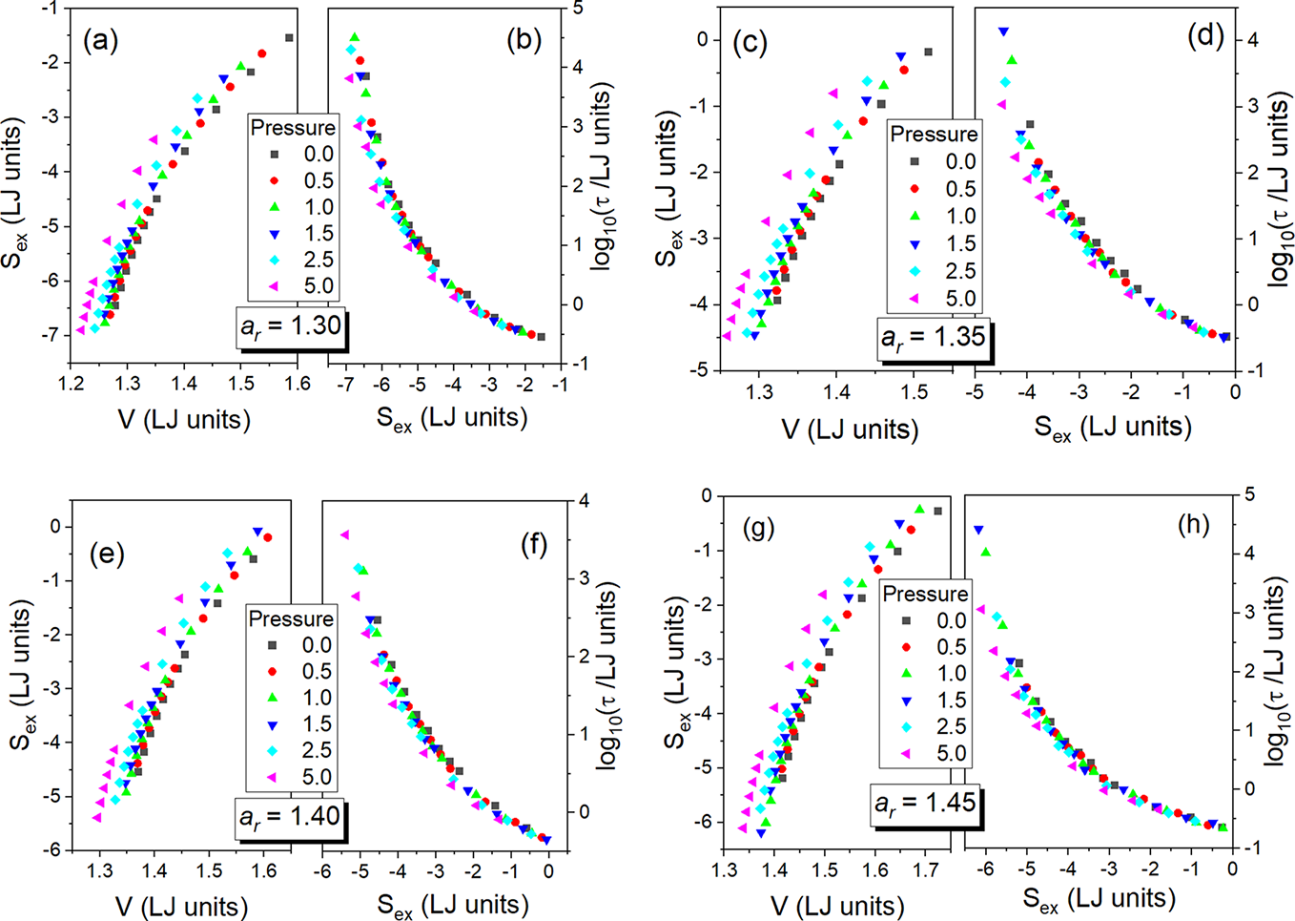
Plots of the excess entropy *S*_ex_ evaluated by using simulation data collected in the supercooled liquid state of the GB model on the assumption of the same reference state as that used to calculate the total system entropy *S* in several isobaric conditions (at pressure *p* = 0, 0.5, 1.0, 1.5, 2.5, and 5.0 in LJ units) and four different anisotropy aspect ratios *a*_r_ = 1.30, 1.35, 1.40, and 1.45 shown in panels (a), (c), (e), and (g), respectively. Failed attempts made at scaling the translational relaxation times τ of the GB supercooled liquid systems with the excess entropy *S*_ex_ for *a*_r_ = 1.30, 1.35, 1.40, and 1.45 are presented in panels (b), (d), (f), and (h), respectively. *V* denotes the particle number volume. Data for τ are taken from ref (18).

In the next step, we can calculate and verify the capabilities of the excess entropy *S*_ex_ to scale the MD timescale in the GB model near the glass transition. Based on the determined values of the total system entropy *S*, we can determine the values of the excess entropy *S*_ex_ = *S* – *S*_id_, assuming that *S*_id_ is the entropy of diatomic ideal gas, which is an appropriate reference to the GB model that consists of unbounded ellipsoidal particles. The volume dependences of the excess entropy are shown in panels (a), (c), (e), and (g) of Figure 3, respectively, in the order of increasing anisotropy aspect ratio *a*_r_ of the examined GB supercooled liquids. Thus, we can test whether *S*_ex_ is able to scale the translational relaxation times τ established for these anisotropic systems in ref (18). However, before all further tests, it should be emphasized that it would be unjustified to use the isomorph theory units in analyzing the data collected from simulations in the *NpT* ensemble, because those units have never been approved for the isothermal–isobaric ensemble.^14,18^ Consequently, we have not employed those units in our analyses presented herein.

As can be seen in panels (b), (d), (f), and (h) of Figure 3, the attempts made herein at excess entropy scaling the translational relaxation times τ earlier determined in ref (18) for the GB supercooled liquid systems have failed in the case of all examined anisotropy aspect ratios, revealing an increasing divergence from the scaling curve with increasing MD timescale τ, i.e., on approaching the glass transition. Hence, one can claim that there is no straightforward relation between τ and *S*_ex_ in this simple anisotropic model of supercooled liquid. Nevertheless, this important finding does not exhaust the issue of the entropy scaling of MD near the glass transition despite the fact that the GB model does not include vibrations and we cannot examine the scaling capability of the configurational entropy.

**Figure 4:**
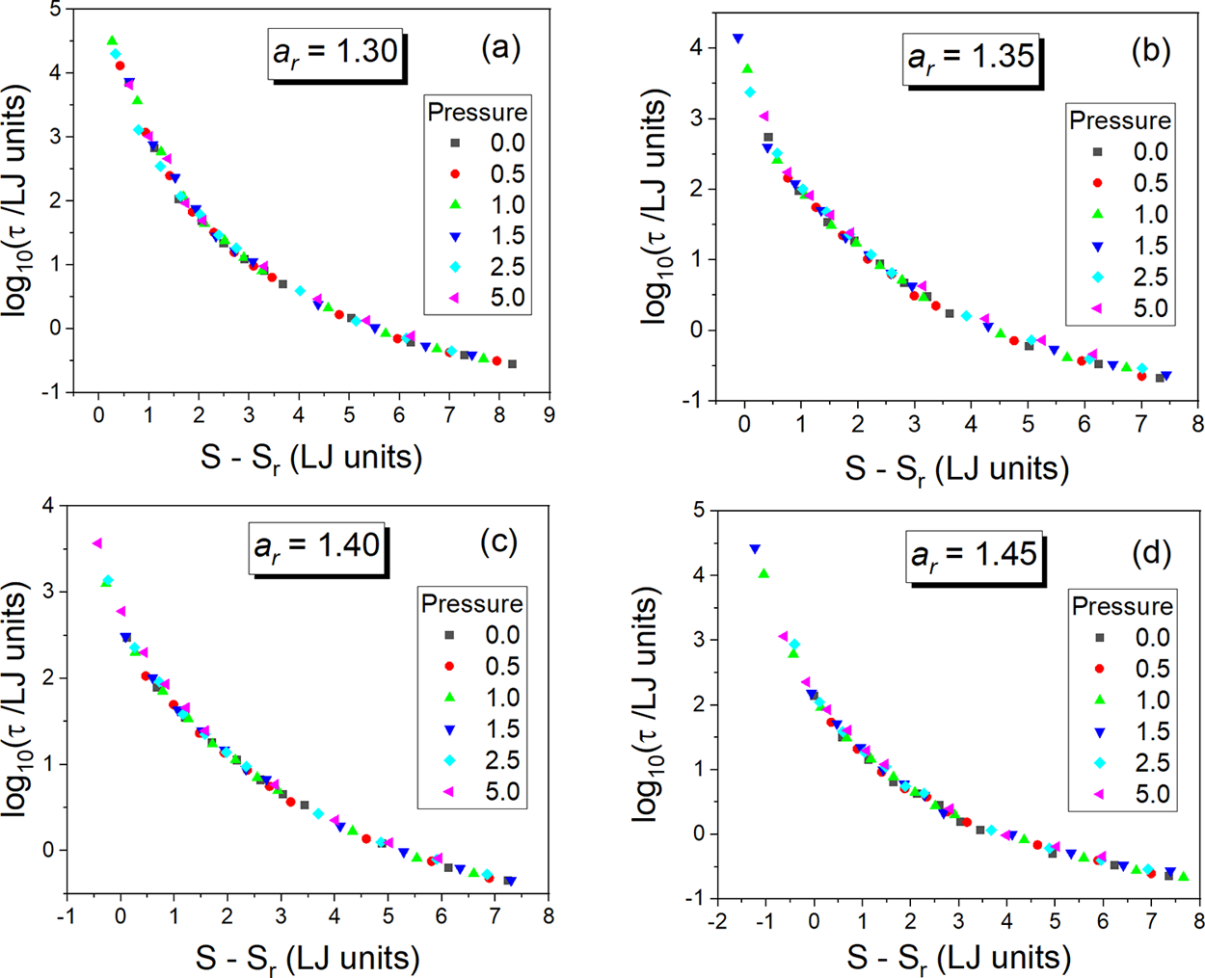
Illustration of the successful scaling of the translational relaxation times τ in the GB supercooled liquid systems with the total system entropy *S* for the anisotropy aspect ratios *a*_r_ = 1.30, 1.35, 1.40, and 1.45 shown in panels (a), (b), (c), and (d), respectively. *V* denotes the particle number volume, whereas *S*_r_*= S*(*T*_g_(*p*_0_),*p*_0_) at zero pressure *p*_0_ = 0. Data for τ are taken from ref (18).

Taking into account all previous research conducted on the total system entropy *S* of molecular supercooled liquids within the density scaling framework by using measurement data,^15,25,26^ one could suspect that *S* in the GB supercooled liquid should obey the power density scaling law, *S* = *f*_s_(ρ^γs^/*T*), but the scaling exponent γ_s_ should be less than γ that enables the density scaling of τ, because γ_s_ was found to be more than two times less than γ for glass-forming materials belonging to different material groups. This additionally poses the question why the MD timescale could not be directly scaled with the total system entropy. However, our results presented in Figure 4 clearly show that τ can be very well scaled with *S* in the GB model in the supercooled liquid state for each examined anisotropy aspect ratio. Seemingly, this result could have been at odds with that experimental outcome. Nevertheless, our further argumentation shows that the total entropy scaling of the translational relaxation times in the explored anisotropic simulation model enables us to gain a better insight into molecular mechanisms that govern the scaling behavior of entropy and MD near the glass transition.

**Figure 5:**
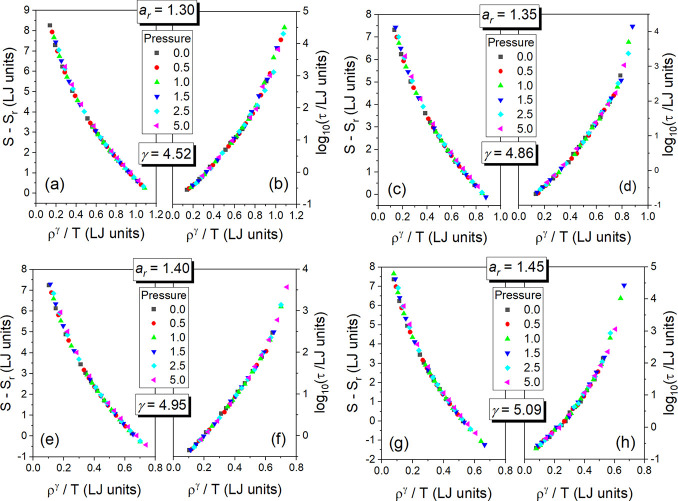
Density scaling plots of the total system entropy *S* in the GB supercooled liquid systems for the anisotropy aspect ratios *a*_r_ = 1.30, 1.35, 1.40, and 1.45 shown in panels (a), (c), (e), and (g), respectively. As assumed to prepare Figures 2 and 4, *S*_r_*= S*(*T*_g_(*p*_0_),*p*_0_) at zero pressure *p*_0_ = 0. For comparison, based on Figure 6 in ref (18), the density scaling plots of the translational relaxation times τ in the GB supercooled liquid systems with the same scaling exponent γ for a given anisotropy aspect ratio *a*_r_ are presented in panels (b), (d), (f), and (h) for *a*_r_ = 1.30, 1.35, 1.40, and 1.45, respectively. Particle number density ρ = *V*^–1^ is used in all panels.

Since we have earlier found^18^ that τ = *f*(ρ^γ^/*T*) and established herein that τ = *h*(*S*) in the GB supercooled liquid, one can suggest that the density scaling law, *S* = *f*_s_(*ρ*^γs^/*T*), is valid for the total system entropy with the scaling exponent, γ_s_ = γ, which is the same as that for the translational relaxation time for a given anisotropy aspect ratio in this model. Indeed, this hypothesis finds its confirmation in the density scaling plots presented for *S* in panels (a), (c), (e), and (g) of Figure 5, which are compared to the density scaling plots for τ in panels (b), (d), (f), and (h) of this figure.

The achieved high quality of both the density scaling behaviors demands of us to consider their possible molecular mechanisms and consequences for our understanding of thermodynamics and MD of the system approaching the glass transition. To do that it is worth invoking the already mentioned interpretation of the configurational entropy, *S*_conf_ = *S* – *S*_vib_, suggested by Johari,^2^ which implies that *S* = *S*_conf_ in the systems without vibrations. The next point consists in the commonly assumed interpretation of the density scaling exponent γ in the case of its invariance for a given material, which is observed for the vast majority of measurement data. This interpretation relates the scaling exponent γ to the exponent of the dominant repulsive term (∼*r*^–3γ^) in an effective intermolecular potential valid for short intermolecular distances *r* in viscous molecular systems. Additionally considering that the molecular vibrations have been typically modeled (in the simplest harmonic case) by a quadratic potential (∼(*r* – *r*_0_)^2^) about an equilibrium position *r*_0_, we may claim that the GB model reveals a nature of the density scaling of entropy near the glass transition, which also relies on molecular interactions. This conception becomes reasonable if we realize that such a quadratic potential is able to reduce the effective potential exponent 3γ in a manner suggested for other potential terms in Supporting Information to ref (27), which explains why γ_S_ < γ if we analyze the experimental data.

## Conclusions

Our analyses of the simulation data collected for the GB model in the supercooled liquid state clearly show that (i) the entropy that is in excess of the ideal gas entropy (*S*_ex_) cannot scale the translational relaxation times τ, whereas (ii) the total system entropy can scale the MD timescale in the GB model and the scaling exponent γ for both *S* and τ is related to the same scaling exponent of the repulsive part of the effective short-range potential suggested to be responsible for the density scaling. By comparing point (ii) to the previous results of the density scaling of *S* determined from measurement data,^15,25,26^ one can conclude that the effective short-range potential that influences the density scaling of the total system entropy *S* is also affected among other interactions by intramolecular vibrations and other intramolecular forces, which are commonly regarded to contribute negligibly small to the effective short-range potential classified as an intermolecular potential that is relevant to the density scaling of MD timescale. However, if a molecular system involved only translational and rotational motions without vibrations and other intramolecular motions, then its thermodynamics and global relaxation dynamics relevant to the glass transition should be governed by the same effective short-range intermolecular potential near the glass transition as established for the GB supercooled liquid. This nontrivial but well-grounded conclusion has been drawn by exploiting the simulation model characterized by a well-defined anisotropy of the molecular shape and the intermolecular potential, involving only the translational and rotational degrees of freedom. Thus, our simple involvement of the molecular anisotropy in MD leads to an entropic linkage between thermodynamics and MD near the glass transition. However, we show that anisotropic models depreciate the importance of the excess-entropy scaling reported by Rosenfeld^28^ based on isotropic potentials, which has been widely applied with varying degrees of success to analyze different anisotropic molecular systems by many authors in the hope of providing a universal scaling picture of molecular phenomena. Instead of this rather artificial method in the case of anisotropic systems, we propose to simply remove the effect of irrelevant interactions on the global MD timescale to achieve a general relation between thermodynamics and global MD. Although the outcome of our findings has not yet considered special interactions such as hydrogen bonds and ionic interactions, which may change the pattern of the density scaling behavior,^4^ it makes much progress toward our better understanding of molecular mechanisms of liquid systems approaching the glass transition and the sought-after universality of their description, consequently increasing a credibility of theorist’s dream^29−31^ to underlie physicochemical properties of liquids via scale-invariant potentials.

## Supporting Information Available

Calculations of entropy values (PDF)
