# Supplementary material for: Entropy Scaling of Molecular Dynamics in a Prototypical Anisotropic Model near the Glass Transition
Source: J Phys Chem B. 2023 May 31;127(23):5334–40. doi: 10.1021/acs.jpcb.3c02429 (PMC10278124; doi:10.1021/acs.jpcb.3c02429)
Supplement: Supplementary file 1 — jp3c02429_si_001.pdf [file jp3c02429_si_001.pdf]

Entropy Scaling of Molecular Dynamics in a Prototypical Anisotropic Model near the Glass Transition

Karol Liszka,[1] Andrzej Grzybowski,[1,*] Katarzyna Grzybowska,[1] Kajetan Koperwas,[1] and Marian Paluch[1]

[1]*Institute of Physics, University of Silesia in Katowice, ul. 75 Pułku Piechoty 1, 41-500 Chorzów, Poland.*

Corresponding Author

* Email: andrzej.grzybowski@us.edu.pl

SUPPORTING INFORMATION

Details of the Calculations of Total System Entropy Values

Following the procedure commonly used in the case of measurement data, to calculate the total system entropy according to its thermodynamic formula given by eq 1 in the main part of the article, we need to know at least the temperature dependence of the isobaric heat capacity C_p(T) at a reference pressure and the temperature-pressure dependences of volume, $V(T,p)$, established from our simulation data collected in this article and our previous paper[1] (cited as ref. 18 in the main part of this article) from the molecular dynamics (MD) simulations in the isothermal-isobaric (NpT) statistical ensemble in the version of the Gay-Berne (GB) model[2]

(cited as ref. 17 in the main part of this article) earlier used by Kapko et al.[3] (cited as ref. 16 in the main part of this article) to simulate the glass transition at zero pressure. It can be clearly seen from the representation, $\Delta S(T,p)=\int_{T_r}^{T} C_p(T,p_r)d\ln T-\int_{p_r}^{p}\left(\partial V(T,p)/\partial T\right)_p dp$, where $\Delta S(T,p)=S(T,p)-S_r$ at a reference state $S_r=S(T_r,p_r)=S(T_g(p_0),p_0)$, which has been assumed by us at the glass transition temperature T_g at zero pressure $p_0 = 0$. Such a reference state (but at ambient pressure) is often applied to calculate thermodynamic functions as entropy or enthalpy by using measurement data.

In the main part of the article, we show that we can linearly approximate the temperature dependence $C_p(T,p_r) = C_1T + C_0$ in the supercooled liquid state in the GB simulation model, which is a common procedure for experimental data obtained from differential scanning calorimetry measurements of various materials near the glass transition. In the main part of the article, there are details of MD simulations performed for four anisotropy aspect ratios a_r = (ellipsoid length/ellipsoid width) = 1.30, 1.35, 1.40, and 1.45 to find the heat capacity data C_p in the GB model near the glass transition at zero pressure $p_r = 0$ as well as values of the parameters C_1 and C_0 of its linear approximation in the supercooled liquid state (see Table 1 in the main part of this aritcle). It is worth noting that the linear representation of $C_p(T,p_r)$ enables to derive analytically the indefinite integral with respect to temperature, which underlies the heat capacity contribution to the total system entropy as follows,

$$\int C_p(T,p_r)d\ln T = C_1T + C_0\ln T + const \tag{S1}$$

However, the pressure-volume-temperature (pVT) data needed to calculate the volumetric contribution to the total system entropy has been established earlier from our MD simulations in the GB model carried out in the NpT ensemble for four anisotropy aspect ratios a_r = 1.30, 1.35, 1.40, and 1.45 near the glass transition that has been detected from the pVT

simulation data. We have explored 6 isobars at pressures p = 0, 0.5, 1.0, 1.5, 2.5, 5.0 in LJ units in the temperature range $0.2 \leq T \leq 0.9$ also in the LJ units by performing the MD simulations in the state points at $T \geq 0.5$ every 0.1 LJ temperature unit and every 0.025 LJ temperature unit at $T < 0.5$ along each isobar, which enabled us to determine accurately the glass transition temperature at each pressure, and then to identify the T-p domain of the supercooled liquid state in the GB model (see Figure 1 in ref. 1 cited as ref. 18 in the main part of this article). To satisfy the thermodynamic conditions in the NpT ensembles, we have used the standard Nosé-Hoover thermostat and barostat, the relaxation times of which have been set to 1.0 and 0.1, respectively. In the MD simulations, a known leap-frog algorithm has been employed at the timestep $\Delta t = 0.001$ in LJ units and the cutoff radius for interactions $r_{cut} = 3.2$ in LJ units. After at least 10^6 timesteps of equilibration runs, the simulation runs appropriate for determining physical quantities used in further analyses ranged from 10^5 to above 10^8 timesteps, depending on the state point. The longer simulations were necessary to achieve reliable results as the liquid approached the glass transition. It is worth noting that we have collected the simulation relaxation data and volumetric data at the same state points, and the equilibrium MD simulations have enabled us to determine properly the translational and rotational relaxation times in the supercooled liquid state in the GB model,[1] which is also briefly descripted in the main part of this article. However, the simulation volumetric data $V(T,p)$ found in the GB supercooled liquid state has been parametrized by using an equation of state (EoS) well designed by some of us[4] (ref. 19 in the main part of this article) to describe pVT data of supercooled liquids,

$$V(T,p) = V(T,p_0)\left[1 + \frac{\gamma_{EOS}}{B_T(p_0)}(p - p_0)\right]^{1/\gamma_{EOS}} , \qquad \text{(S2)}$$

where the exponent γ_{EoS} is a fitting parameter, and the following temperature functions are applied to parametrize the volume and isothermal bulk modulus at the state points (T,p_0),

$V(T,p_0)=A_0+A_1(T-T_0)+A_2(T-T_0)^2$ and $B_T(p_0)=b_0\exp\left[-b_1(T-T_0)\right]$, where $b_0=B_{T_0}(p_0)$, $b_1=-\partial\ln B_T(T,p_0)/\partial T\big|_{T=T_0}$, $A_0=V(T_0,p_0)$, $A_1=\partial V(T,p_0)/\partial T\big|_{T=T_0}$, $A_1=(1/2)\partial^2V(T,p_0)/\partial T^2\big|_{T=T_0}$ are fitting parameters, and (T_0,p_0) is a fixed reference state point chosen at p_0=*0* and the glass transition temperatures, $T_0=T_g\left(p_0\right)$. By fitting the simulation temperature-pressure dependences of the particle number volume to eq S2, we have established the values of the EoS fitting parameters,[1] which have been collected in Table 1 in ref. 1 cited as ref. 18 in the main part of this article, where the same symbols of the EoS parameters have been used as those in eq S2.

As shown in eq S1, one can easily calculate analytically the indefinite integral with respect to temperature, which contributes to the total system entropy. Although the EoS given by eq S2 is relatively complicated, it has turned out that the other contribution to the total system entropy *S*, which is the indefinite integral with respect to pressure, $\int\left(\partial V(T,p)/\partial T\right)_p dp$, can be also derived analytically as follows,

$$\left(\partial V(T,p)/\partial T\right)_p=\left[1+\frac{\gamma_{EOS}\exp\left[b_1(T-T_0)\right]}{b_0}\left(p-p_0\right)\right]^{-1/\gamma_{EOS}}\left\{A_1+2A_2\left(T-T_0\right)\right.$$
$$\left.-\frac{\left(A_0+A_1(T-T_0)+A_2(T-T_0)^2\right)b_1\exp\left[b_1(T-T_0)\right]}{b_0+\gamma_{EOS}\left(p-p_0\right)\exp\left[b_1(T-T_0)\right]}\left(p-p_0\right)\right\} \tag{S3}$$

$$\int\left(\partial V(T,p)/\partial T\right)_p dp=-\frac{\exp\left(-b_1T\right)}{\gamma_{EOS}-1}\left[1+\frac{\gamma_{EOS}\exp\left[b_1(T-T_0)\right]}{b_0}\left(p-p_0\right)\right]^{-1/\gamma_{EOS}}$$

$$\times\left\{A_0b_1\left(b_0\exp\left(b_1T_0\right)+\left(p-p_0\right)\exp\left(b_1T\right)\right)\right.$$

$$-A_1\left[\left(p-p_0\right)\left(\gamma_{EOS}-b_1\left(T-T_0\right)\right)\exp\left(b_1T\right)+b_0\left(1-b_1\left(T-T_0\right)\right)\exp\left(b_1T_0\right)\right]$$

$$\left.-A_2\left(T-T_0\right)\left[\left(2\gamma_{EOS}-b_1\left(T-T_0\right)\right)\left(p-p_0\right)\exp\left(b_1T\right)+b_0\left(2-b_1\left(T-T_0\right)\right)\exp\left(b_1T_0\right)\right]\right\} \quad \text{(S4)}$$

The analytical formula for the total system entropy S enhances the accuracy of our analyses that rely on both the total system entropy and the excess entropy S_{ex}, because the latter is defined by the difference, $S_{ex} = S - S_{id}$, where S_{id} has been assumed herein as the entropy of the diatomic ideal gas. Such an ideal gas constitutes the appropriate reference compared to the ellipsoidal particles of the GB model, which has been argued in the main part of this article.

REFERENCES

(1) Liszka, K.; Grzybowski, A.; Koperwas, K.; Paluch, M. Density Scaling of Translational and Rotational Molecular Dynamics in a Simple Ellipsoidal Model near the Glass Transition. *Int. J. Mol. Sci.* **2022**, *23*, 4546, DOI:10.3390/ijms23094546.

(2) Gay, J. G.; Berne, B. J. Modification of the overlap potential to mimic a linear site-site potential. *J. Chem. Phys.* **1981**, *74*, 3316-3319, DOI:10.1063/1.441483.

(3) Kapko, V.; Zhao, Z.; Matyushov, D. V.; Angell, C. A. “Ideal glassformers” vs “ideal glasses”: Studies of crystal-free routes to the glassy state by “potential tuning” molecular dynamics, and laboratory calorimetry. *J. Chem. Phys.* **2013**, *138*, 12A549, DOI: 10.1063/1.4794787

(4) Grzybowski, A.; Grzybowska, K.; Paluch, M.; Swiety, A.; Koperwas, K. Density scaling in viscous systems near the glass transition. *Phys. Rev. E* **2011**, *83*, 041505, DOI:10.1103/PhysRevE.83.041505.
